# Usefulness of occupation and industry information in mortality data in South Africa from 2006 to 2015

**DOI:** 10.1186/s12889-019-7177-3

**Published:** 2019-07-03

**Authors:** Kerry S Wilson, Nisha Naicker, Tahira Kootbodien, Vusi Ntlebi, Felix Made, Nonhlanhla Tlotleng

**Affiliations:** 1 National Institute for Occupational Health, National Health Laboratory Service, Johannesburg, South Africa; 2 School of Public Health, Faculty of Health Sciences, University of the Witwatersrand, Johannesburg, South Africa; 3 Environmental Health Department, Faculty of Health Sciences, University of Johannesburg, Johannesburg, South Africa

**Keywords:** Mortality, Pneumoconiosis, Mining, Manufacturing

## Abstract

**Background:**

There is no population based occupational health surveillance system in South Africa, thus mortality data may be a cost effective means of monitoring trends and possible associations with occupation. The aim of this study was to use deaths due to pneumoconiosis (a known occupational disease) to determine if the South African mortality data are a valid data source for occupational health surveillance in South Africa.

**Methods:**

Proportions of complete occupation and industry information for the years 2006–2015 were calculated for working age and retired adults. Deaths due to pneumoconiosis were identified in the data set and mortality odds ratios calculated for specific occupations and industry in reference to those who reported being unemployed using logistic regression.

**Results:**

Only 16.1% of death notifications provided a usual occupation despite 43.1% of the population being employed in the year. The MORs for occupation provided significant increased odds of pneumoconiosis for miners (9.04), those involved in manufacturing (4.77), engineers and machinery mechanics (6.85). Along with these jobs the Mining (9.8), Manufacture (2.2) and Maintenance and repair industries (6.0) have significantly increased odds of pneumoconiosis deaths. The data can be said to provide a useful source of occupational disease information for surveillance where active surveillance systems do not exist.

**Conclusion:**

The findings indicate valid associations were found between occupational disease and expected jobs and industry. The most useful data are from 2013 onwards due to more detailed coding of occupation and industry.

## Background

National occupational health surveillance is a necessary and key part of an effective National Occupational Health Programme. These surveillance results can be used to determine the burden of known and suspected occupational diseases and injuries and identify emerging hazardous workplaces or jobs. A surveillance system supports prevention and control of occupational hazards along with evaluation of controls. Surveillance can also play a role in the identification of possible causal agents, along with evaluating the efficacy of interventions for prevention of disease. A clear picture of occupational disease and injuries in South Africa is needed to make best use of limited resources to improve worker’s health. This will in turn reduce the burden on South African public health resources by describing industries who should be contributing to treatments and preventing occupational disease and injuries. This information is important for setting effective policies and identifying priorities in controls programs and for creating an equitable responsive compensation program when prevention fails. Currently there is no population-based occupational health surveillance system in South Africa, which provides public reports or covers all workers, formal and informal. Mortality data with information on the usual occupation during the life of the deceased may be a useful proxy for an occupational health and safety surveillance system.

Mortality data are collected by many governments around the world and provides useful population-level data on underlying causes of death that are almost impossible to collect regularly in any other way. Although there are many limitations to death registry data, including missing data and over counted deaths and misclassification, but this data remains a cost-effective useful source of information [1]. The advantages of using death registry data include the size of the database and the complete coverage of South Africa. The National Institute for Occupational Health and Safety (NIOSH) in the USA uses mortality data for their National Occupational Mortality Surveillance (NOMS) [2]. They identified death registry data as a source of surveillance of occupational disease by industry and job type [3]. In South Africa the form DHA-1663 is completed on the death of a citizen. This form requests usual occupation and usual industry during their lifetime not at the time of death.

In this paper, pneumoconiosis which is a rare but mostly occupational disease is used as a case study to investigate the validity of the associations between cause of death and usual occupation and industry in the mortality data. Pneumoconiosis is an interstitial lung disease resulting from dust exposure. There are a number of forms of pneumoconiosis, each caused by a different type of dust [4]. Coal Worker’s Pneumoconiosis (CWP), asbestosis, mixed dust pneumoconiosis, and silicosis are all examples of pneumoconiosis due to different occupational exposures. Pneumoconiosis occurs mainly in occupational settings using high-energy processes that generate and release respirable size particles [5, 6]. It is a progressive fibrotic lung disease, which can be chronic or acute and can be detrimental in the long term [7, 8]. Historically, the South African gold mining industry has had many years of experience with silicosis a type of pneumoconiosis [9]. Other occupational groups associated with pneumoconiosis are construction workers, electricians, truck drivers, farmers and operating engineers [10]. This study proposed to use the 2006–2015 mortality data to determine trends in reporting of occupation and to determine the mortality odds ratios (MORs) for occupation and industry with pneumoconiosis.

## Methods

Mortality, usual occupation and industry data as captured from death certificates in South Africa from 2006 to 2015 were retrieved from Statistics South Africa [11]. This is the government bureau responsible for the collection, production and dissemination of official and other statistics, and for the conducting of census in the country. The data obtained from Stats SA included deaths due to all causes, age, sex, education occupation and industry. The underlying cause of death, which was completed by the person responsible for certifying the death and then coded by the Stats SA using the 10th International Classification of Diseases Codes (ICD10), was used to calculate proportions of death in the cause of death groups for each occupational group. Occupation group was also coded by Stats SA staff using, the South African Standard Classification of Occupations (SASCO) list in Table 1 [12]. The SASCO list was based on the United Nations international standard classification of occupations (ISCO) to allow for comparisons between countries [13].

**Table 1:** Provides the ten occupational groups used by Statistics South Africa along with the 10 industry groups

No.	Occupation^a^	Includes	No.	Industry 2006–2010
0	Armed forces, occupations unspecified a not elsewhere classified	Unemployed and armed forces and those forms with blank spaces	i	Private households, exterritorial organ
1	Legislators, senior officials and managers	CEOs, senior officials, managers of all occupations	ii	Agriculture, hunting, forestry and fish
2	Professionals	Science, engineering, health, teaching, business, information and communication, legal, cultural and social professionals	iii	Mining and quarrying
3	Technicians and Associate professionals	Associate professionals in the same areas as group 2	iv	Manufacturing
4	Clerks	General, keyboard, customer services numerical and material recording and clerical support workers	v	Electricity, gas and water supply
5	Service workers, shop and market sales	Personal services, Sales workers, Protective services and armed forces	vi	Construction
6	Skilled agricultural and fishery worker	Market orientated agricultural, forestry, and fishing, along with subsistence farmers, fishers and hunters.	vii	Wholesale and retail trade; repair of m
7	Craft and related trade workers.	Building and trades, metal, machinery and related, handcraft, printing, electrical and food processing, woodworking and garment workers	viii	Transport, storage and communication
8	Plant and machine operators and assemblers	Stationary plant and machine operators, assemblers, drivers and mobile plant operators	ix	Financial intermediation, insurance, re
9	Elementary occupations	Cleaners and helpers, agricultural labour, labour in mining, construction, manufacture, food preparation, street sales, refuse workers and other	x	Community, social and personal services
			97	Unknown
			98	Not applicable
			99	Unspecified and other activities not adequately defined

^a^sub and minor occupation categories are listed in the South African Standard Classification of Occupation 2012 [12]

It is a legal requirement in South Africa for the form DHA-1663 (the South African notice of death/stillbirth) is completed on the death of a citizen. This form collects important information about the decedent including age, sex, date of death, and information on the circumstances and cause of death. The Department of Home Affairs then issues a death certificate and burial order. The DHA-1663 form contains six sections (A to G), each of which is required to be completed and checked by various approved personnel. On page one of the death notification form DHA-1663A part A, question 19 asks the open question “what was the usual occupation of the deceased (the type of work done during most of life)” and Question 20 then asks an open question on the type of business or industry. This analysis is limited to 2006–2015 (Table 7 in Appenidx) as in the years 1997–2005 occupation was not coded, it was seen as too poorly completed, and the value of coding the data was questioned. Recently data from the years 1997–2015 has been re-released with consistent coding for major occupation group. No sub or minor occupation group information was released. Thus we relied on the individual year data as provided previously by Statistics South Africa.

### Data management

Deaths from 2006 to 2015 were used to determine mortality rates, coding of occupation and industry and reporting of occupation. In the coding of the South African mortality data, similar jobs were grouped into ten main groups (Table 1), but many of these occupations while similar have different exposures. Thus, the value of the ten occupational groups is limited for longitudinal and investigative analysis. For example, elementary worker – the largest employment group - encompasses street workers, cleaners, food preparation assistants, mining and building labourers. This limits the usefulness of mortality data for occupational disease surveillance and investigations. The years from 2013 to 2015 contain more disaggregated occupational information in two extra variables, sub occupation and minor occupation allowing for surveillance investigations. Industry was coded in 9 groups until 2010 when more disaggregated industries were coded. These are useful particularly in conjunction with occupation providing useful information on workplace exposures. Due to the coding of occupation before 2013 not containing the three digits codes, only 2013–2015 data which contained the more detailed information were used to investigate the association between cause of death, usual occupation and industry. It is accepted that South African mortality data are of mediocre quality due to a range of factors including limited resources [14, 15]. The variables used in the analysis were cleaned by recoding any unknown values to missing in age year, marital status, education and sex; so that the numbers used to code this information are not included in calculations. All deaths aged 14 and below were removed from the data set as this analysis focuses on occupational causes of disease (15 years and above is considered working age). Marital status was condensed into ever married and never married. Minor occupation (coded to three digits) was used, but where there was only one death due to pneumoconiosis in the three years, the occupation was combined with a similar one. Legislators and senior officials were combined with managing directors and CEOs due to the similar nature of their occupations in terms of hazards, building frame workers and building finishers were combined. Combining these occupational and industry groups involved adding the cases and non-cases from each group together to create one new group. A similar exercise was performed with industry where the central government was combined with other services, and non-specified retail was combined with other retail. These changes reduced wide confidence intervals by increasing numbers in each group, but the trends remained the same.

### Statistical analysis

Mortality odds ratios were chosen over standardised mortality rates as the number of workers in each occupation for each year in South Africa is mostly unknown. Also due to the poor completion of the occupation question in the death certificate, it is not possible to determine the proportion of workers who worked in each occupation or industry who had their information recorded on the death notification. Data analysis was conducted using STATA software version 14.2 and Microsoft Excel spreadsheet 2010. Reported usual job and industry in deaths over age fifteen years were used to determine MOR’s. Trends in reporting of occupation and industry were created and illustrated in graphs. The 2010 SA NBD list, which comprises 140 specific causes of death was used to group pneumoconiosis deaths, these are ICD 10 codes J60 to J65. Due to the small numbers of deaths due to pneumoconiosis data from the years 2013–2015 were combined. They are the only years available with coding to three digits for occupation. Combining years will improve the analysis by increasing the power and remove spurious associations.

MORs were calculated using logistic regression of deaths due to pneumoconiosis against deaths due to all other causes. The outcome chosen here, pneumoconiosis, has a low event rate. However, we could not apply Firth’s penalised maximum likelihood estimation for the regression analysis logistic regression although it is the recommended method for analysing rare events in large datasets to reduce bias as we did not have the required computing power [16]. The MOR’s determined were adjusted for age, sex, smoking and death year all significant factors associated with annual mortality. We compared all occupation minor-groups to persons not economically active to adjust for socioeconomic deprivation.

## Results

### Mortality data

**Fig. 1:**
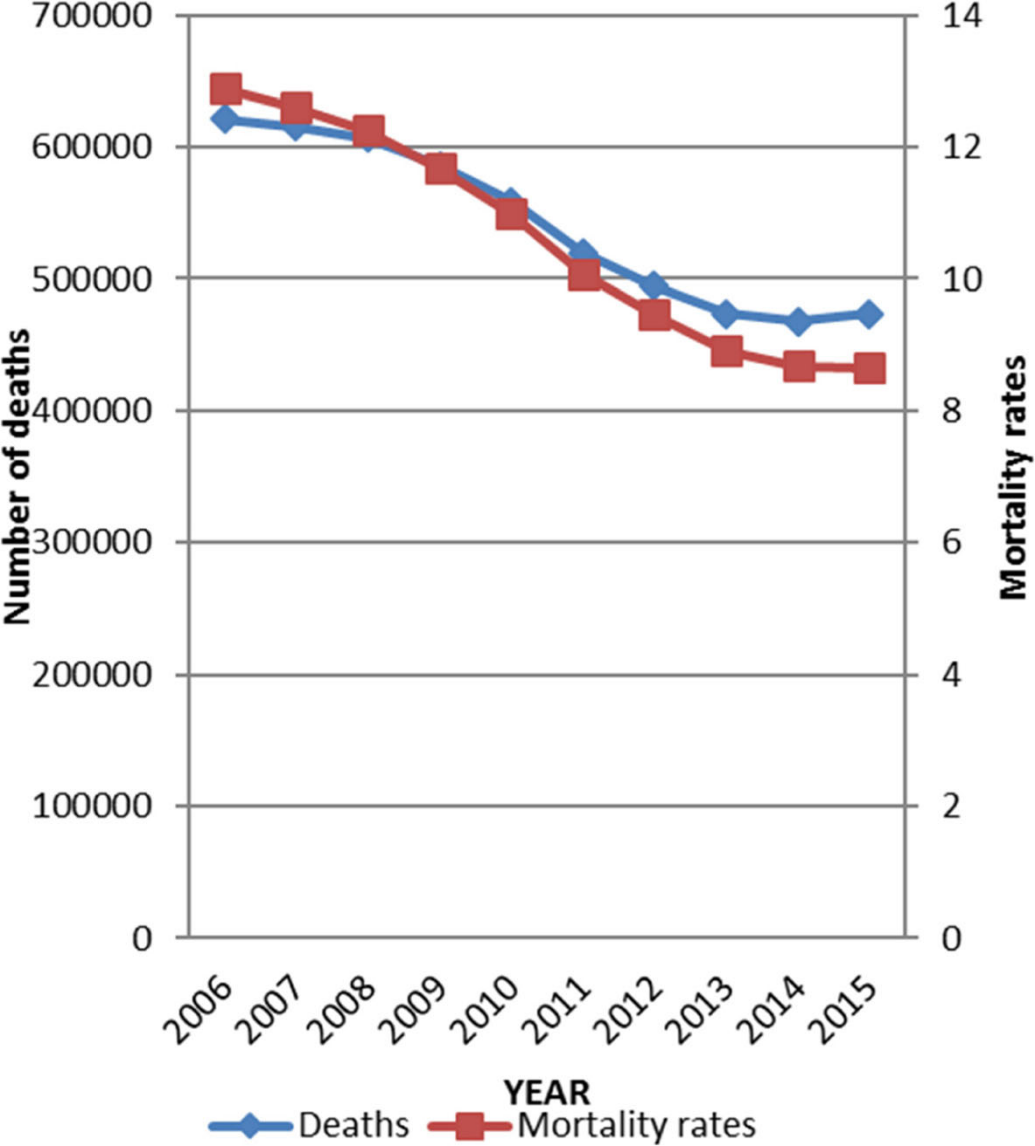
Graph of absolute mortality and mortality rates for South Africa from 2006 to 2015 [taken from Statistics South Africa (Stats SA, [11]

Mortality in South Africa from 2006 to 2015 showed an absolute decrease from 621,378 deaths in 2006 to 473,938 deaths in 2015 while the overall crude rates also decreased (Fig. 1).

**Fig. 2:**
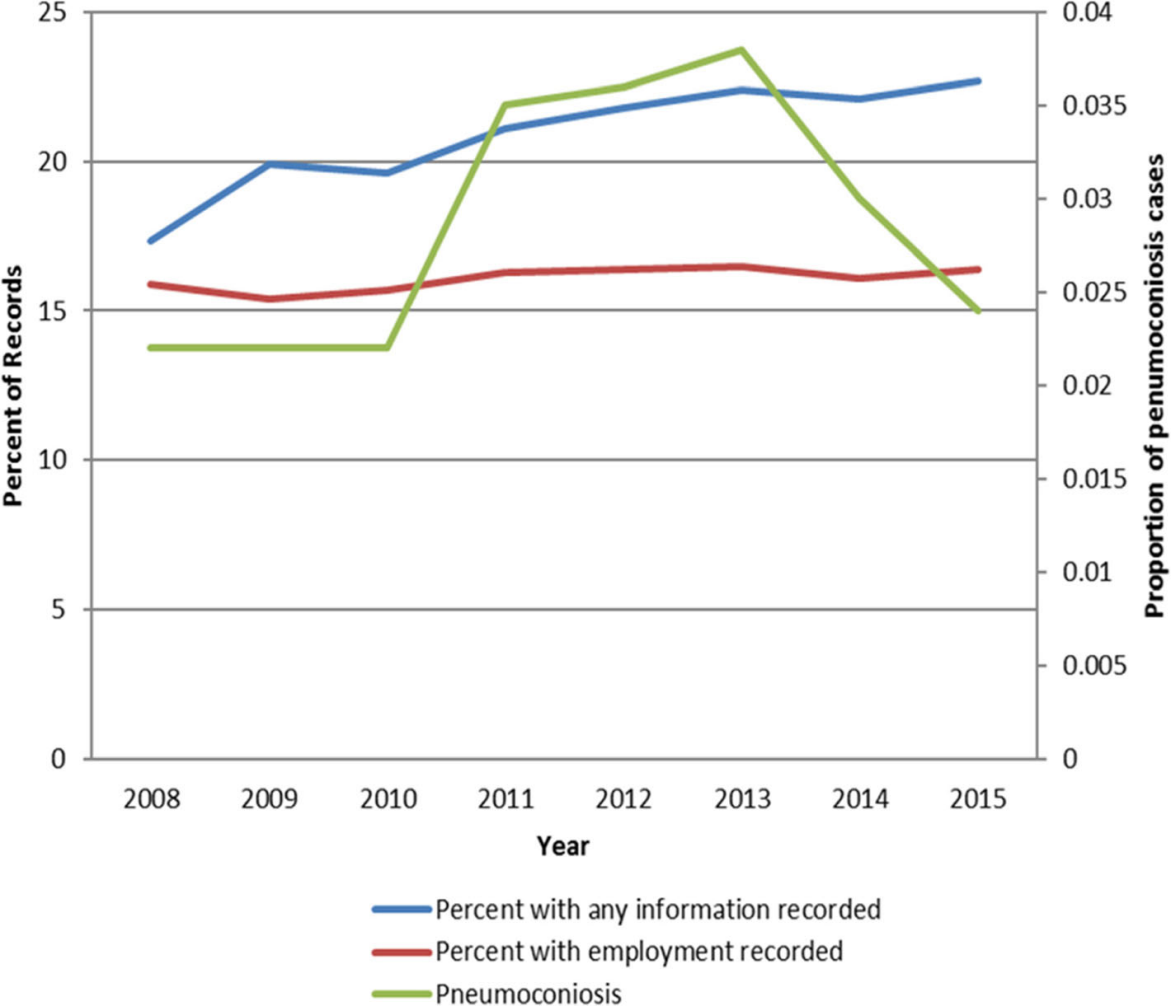
Graph of the proportion of records > 15 yrs. with any information on usual occupation and industry information provided including unemployed

Only a small proportion of death notification forms (mean 16.1%) of those of working age (15–65) were completed with usual occupation and industry information (Fig. 2), while 43.1% of the same age population were employed at the time [17]. Risk of death may not be the same between employed and unemployed. The labour force surveys (LFS) were started by Statistics South Africa in 2008. Therefore information for 2006 and 2007 is not available.

The proportion of those completing the occupation question has increased by 5% over the past eight years while those reporting actual employment has changed little with an average of 16% of deaths between the ages 20 and 65 containing information of usual employment from 2008 to 2015 (Table 2). The increase in the proportion reporting some employment increased significantly by 0.5% from 2008 to 2015 (proportions test *p* = 0.0337), but the increase consisted mostly of people reporting being unemployed.

**Table 2:** The proportion of forms completed with any occupation information for ages 20–65 years

	2008	2009	2010	2011	2012	2013	2014	2015
Proportion completing the usual occupation question (includes unemployed)	17.3	19.9	19.6	21.1	21.8	22.4	22.1	22.7
Proportion who reported being employed	15.9	15.4	15.7	16.3	16.4	16.5	16.1	16.4
LFS proportion employed	45.9	43.9	41.8	41.9	42.2	42.7	42.8	43.7

*Taken from Stats SA [11]

The ten categories used to group occupations have remained the same over these ten years although the location of the deaths of people who worked for the armed forces moved within categories. They were placed in group 0 for 2006–2012 excluding 2008 where they had their own group and then placed in group 5 for 2013 to 2015. This makes an analysis of the trends in cause of death by occupational group difficult. The subcategories and minor occupation groups provide more useful data for analysis as different occupations are clearly defined [16].

### Pneumoconiosis in 2013–2015

The most recent three years of mortality data were combined in a total of 1,363,392 deaths which provided 450 deaths due to pneumoconiosis from 2013 to 2015. A proportion of only 26.3% of the pneumoconiosis deaths were in females.

The most common reported pneumoconiosis was Unspecified although this group appears to contain some misclassified deaths as the proportion of female deaths is much higher than in the other types and the main province of death was not a mining centre nor a well-recognised labour-sending area of South Africa [18]. Those with pneumoconiosis and TB appeared to die at a younger age while those with asbestos and mineral fibres exposure died at a later stage as seen in Table 3.

**Table 3:** Types of Pneumoconiosis and their proportions in South African deaths

Pneumoconiosis type	*n* (%)	Male %	Most common province	Most common occupation^a^	Median age yrs
Coal worker’s	9 (2)	75	Gauteng	Mining	62
Asbestos and mineral fibres	152 (34)	83	Northern Cape	Elementary work	74
Dust with silica	63 (14)	95	Free State	Metal Processing	61
Inorganic dust	0				
Unspecified	182 (40)	53	Kwa Zulu Natal	Mining	68
With TB	44 (10)	93	North West	Mining	57
Total	450	79			64

^a^Excluding unspecified and pensioners

There are a number of significant sociodemographic and occupation differences observed between those with pneumoconiosis and those with other causes of death, using chi-square analysis (Table 4). These are likely to be factors associated with working in a dusty job or industry rather than pneumoconiosis itself.

**Table 4:** Description of demographic data in pneumoconiosis deaths compared to other causes of death

	Pneumoconiosis (*n* = 452) %	Other causes (*n* = 1,362,940) %	*P* value
Sex (Male)	73.6	52.3	< 0.001
*Death Year*			< 0.001
2013	44.5	33.3	
2014	31.0	32.9	
2015	24.6	33.7	
*Marital status*			< 0.001
Never married	25.9	52.2	
Married	55.8	32.1	
Divorced or Widowed	18.2	15.7	
*Smoking*			0.017
Non smoker	19.3	18.6	
Smoker	46.4	40.8	
unknown	34.2	40.6	
*Education*			0.017
None	19.9	16.7	
Any primary school	41.1	33.9	
Any high school	36.0	45.7	
Tertiary	3.0	3.7	
*Province of Death*			< 0.001
Eastern Cape	13.3	14.9	
Free State	7.9	7.2	
Gauteng	15.8	21.3	
KwaZulu-Natal	10.0	17.7	
Limpopo	13.1	10.1	
Mpumalanga	5.6	7.5	
Northern Cape	12.2	3.0	
North West	9.5	7.5	
Western Cape	12	10.7	
Outside SA	0.7	0.2	
Mean Age (SD)	68.(12.9)	56.(20)	< 0.001
*Occupation Group*			< 0.001
Occupation unspecified	78.9	85.5	
Legislators and senior officials	1.1	0.5	
Professionals	2.0	1.7	
Technicians and associate professionals	1.3	0.6	
Clerks	0.2	0.6	
Service Workers	1.1	1.7	
Skilled agricultural	1.1	0.8	
Craft and trade	2.7	1.5	
Plant and Machine operators	6.0	1.8	
Elementary occupation	5.6	5.2	

These differences were then adjusted for in a logistic regression to identify those industries with increased odds of dying of pneumoconiosis (Table 5).

**Table 5:** Industry association with pneumoconiosis deaths 2013–2015

Industry	Other causes *n*	Pneumoconiosis *n*	Adjusted MOR^a^	95% CI
Private households	32,710	9	1.06	0.53–2.12
Not economically active	187,865	86	ref	
Unemployed people; people seeking work	177,999	35	0.97	0.64–1.47
Unspecified activities	656,630	204	1.05	0.79–1.37
Other activities not adequately defined	48,361	14	1.10	0.62–1.95
Growing of crops; market gardening; horticulture	874	8	0.85	0.41–1.78
Mining and quarrying	13,241	56	9.83	6.92–13.96
Manufacture	9339	10	2.27	1.28–4.79
Production; collection and distribution of electricity	4043	3	1.75	0.55–5.54
Building constructions; civil engineering	14,287	4	0.72	0.26–1.96
retail trade in new goods in specialised stores or retail stores	14,159	6	1.24	0.54–2.85
Maintenance and repair of motor vehicles	777	2	6.00	1.47–24.55
Other land transport	11,144	6	1.39	0.60–3.19
Business activities n.e.c.	5964	2	0.84	0.21–3.42
Educational services	8911	2	0.71	0.17–2.90
Other service activities	13,254	3	0.37	0.12–1.16
Total	1,232,299	450		

^a^adjusted for Death year, smoking, age and sex

Only three industries showed significantly increased MORs for pneumoconiosis: Mining, manufacturing and maintenance of vehicles (Table 5). No industries show a protective effect although there were a number of industries with no cases of pneumoconiosis suggesting they are protective (not shown here). Mining accounted for the largest number of pneumoconiosis deaths where industry was specified.

**Fig. 3:**
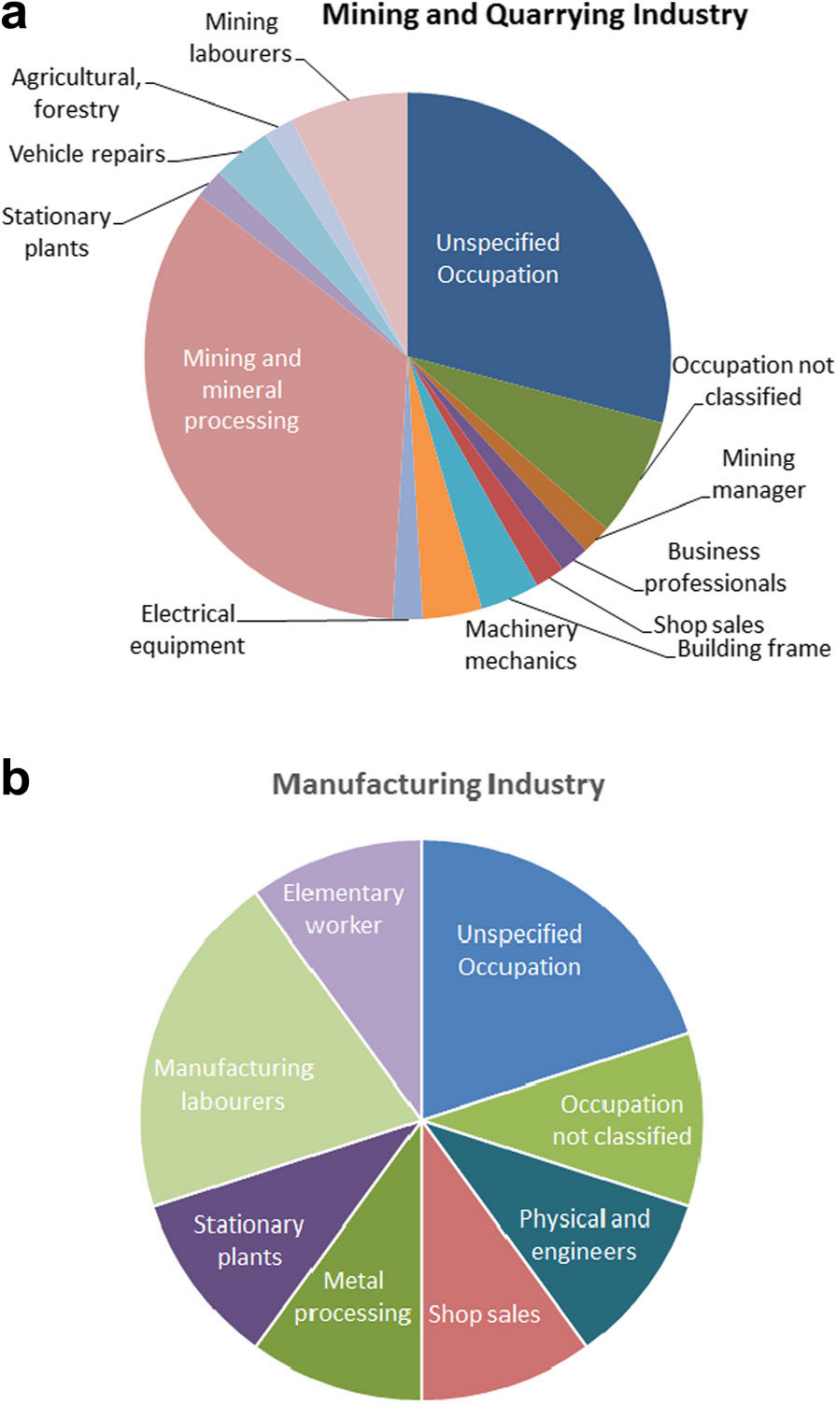
Investigation of occupations with pneumoconiosis deaths within the reported industries. **a** Mining industry significantly increased MORs and reported occupations. **b** Manufacturing industry also had significantly increased odds of pneumoconiosis and reported occupations

Nine occupational groups had significantly increased odds of pneumoconiosis death (Table 6). When industry was analysed most of these were related to the mining or manufacturing industries despite their job titles (Fig. 3). A number of occupations had non-significantly increased odds. Some of these may be due to small numbers such as mining and construction managers.

**Table 6:** Minor occupations association with pneumoconiosis deaths 2013–2015

Occupation	Other causes *n*	Pneumoconiosis *n*	Adjusted MOR^a^	95% CI
Unspecified Occupation	623,831	210	1.21	0.83–1.77
Unemployed persons	179,485	35	ref	
Occupation not elsewhere classified	25,922	9	1.25	0.60–2.61
Occupation not adequately defined	50,021	16	1.19	0.66–2.16
Pensioners and other	161,998	81	1.07	0.71–1.62
Not economically active	15,233	4	0.93	0.33–2.67
Legislators and Managing directors and CEOs	1200	2	3.15	0.75–13.18
Mining construction and distribution managers	334	1	5.86	0.79–43.12
Other Services managers	3379	2	1.44	0.35–6.01
Electro technology Engineers	511	2	6.85	1.63–28.77
Other teaching professionals	7428	2	0.92	0.22–3.87
Business and Admin Professionals	3702	5	3.04	1.18–7.82
Physical and engineering technicians	1111	4	8.48	2.99–24.03
Mining, manufacturing construction supervisors	467	1	5.06	0.68–37.13
Nursing and midwives associate professionals	450	1	9.22	1.25–67.81
Shop sales	7364	4	2.05	0.72–5.79
Protective services workers	10,680	2	0.68	0.16–2.86
Subsistence farmers	2524	4	2.36	0.83–6.72
Building frame and finishers	6672	5	1.77	0.69–4.55
Machinery mechanics, Blacksmiths and toolmakers	4620	5	2.63	1.02–27.92
Electrical equipment installers	2302	2	2.09	0.50–8.74
Mining and mineral processing	5113	20	9.04	5.17–15.79
Metal Processing plant operators	721	2	6.67	1.60–27.92
Other stationary plant operators	3356	2	1.82	0.43–7.60
Car, van and motorcycle drivers	10,628	3	0.83	0.25–2.71
Domestic hotel and offices cleaners	25,153	4	0.77	0.27–2.20
Agricultural, forestry and fishery workers	10,306	4	1.02	3.63–2.90
Mining and construction labourers	2105	4	5.36	1.90–15.15
Manufacturing labourers	1322	2	4.77	1.14–19.88
Other elementary work	31,162	12	1.23	0.64–2.38
Total		450		

^a^Logistic regression adjusted for sex, age, smoking and year of death

The combination of occupation and industry provides some useful information such as the number of non-core occupations at risk within an industry, and the possible high-risk occupations within the industry (Fig. 3). In a number of deaths, information is only provided on one question either occupation or industry but not both. Combining them increases the information available. Some occupations are industry-specific while others are not and providing the industry those jobs were associated with, provides exposure information. For example, vehicle repairs and mechanical maintenance jobs can be found in many industries. However, only in mining and manufacturing are they at risk of pneumoconiosis. One pneumoconiosis death reported a nursing occupation. This case may have been a misclassified TB case as TB may be confused with pneumoconiosis, or it could represent a nurse employed on a mine. This case will require further investigation [19].

Figure 3 suggests that many unexpected jobs in the mining and manufacturing industries are at risk of dying of pneumoconiosis such as shop sales people and business and administration, not only those commonly associated with pneumoconiosis such as mining professionals and labourers although these do form the largest groups. In the manufacturing industry, there were fewer types of jobs at risk compared to the mining industry. Of further interest in the value of the data and possible improvement, pensioners formed the largest part (94%) of the non-economically active group at risk of pneumoconiosis illustrating the value of collecting correct data. The question is “What was the person’s usual occupation?” not “What was the current occupation?” This means pensioner is not a valid answer.

## Discussion

Reliable estimates of the burden of disease and injury due to occupation are crucial for determining national health and labour priorities. These may come from a population-based surveillance system, but in resource-limited health settings, there are many competing interests for funds, so other data sources may be used. The mortality registry data where occupational and industry information is collected is a possible auxiliary source of occupational health and safety surveillance data.

Mortality data are available for the entire population, and the South African mortality data are considered of medium level quality based on the WHO (World Health Organization) criteria [14]. In a study by Joubert et al., the South African national vital registry mortality statistics were “rated satisfactory for coverage and completeness of death registration, temporal consistency, age/sex classification, timeliness, and sub-national availability” [15]. The use of mortality data removes the expense of collecting primary data and does not require the mandating and enforcing of reporting causes of morbidity and mortality. Mortality data of medium quality has been used internationally for a similar purpose with reliable results [10, 14]. The data can be improved, and this should be on the agenda of those responsible for collecting the data.

This paper aimed to demonstrate the usefulness of the mortality data for occupational disease and risk surveillance through the association of pneumoconiosis mortality to known occupations and industries. The 450 pneumoconiosis deaths found in the combined data set are most likely an under-reported number, an international study comparing hospital discharge files and cause of death found pneumoconiosis was more likely to be reported as a contributory factor rather than the underlying cause of death [10, 20]. In addition, as the diagnosis of pneumoconiosis is complicated, it requires autopsy of the lungs after death or an x-ray diagnosis during life, death due to pneumoconiosis is likely to be under-recognized particularly in rural areas. There is also a chance for a misdiagnosis of pneumoconiosis were the changes seen in the lung were due to other causes such as lesions due to infectious disease such as tuberculosis, and with carcinomas [19].

The difficulty of diagnosing pneumoconiosis is evident in the mortality data used in this study where the largest number of pneumoconiosis cases were classified as unspecified type and included a much larger proportion of women than the specified pneumoconiosis. Also, the larger proportion of unspecified type cases came from a province of South Africa with much less mining than the four main mining provinces which form the main provinces in the specified cases. This limitation may come from non-medical or poorly trained staff certifying of the cause of death. Despite this limitation, the data still provided plausible associations and thus remains useful as a surveillance tool.

We observed a clear association between the increased odds of dying from pneumoconiosis and working as a miner or having worked in the mining industry. This is supported by a large body of work identifying mining as a major risk factor for pneumoconiosis. A total of 142 review papers from 1964 to 2018 on the topic in the Medline database Pub Med were found. Other industries with significantly increased odds in the mortality data were manufacturing and maintenance and repair of motor vehicles. Unfortunately, the types of manufacturing were not specified in the mortality data. Increasingly pneumoconiosis is being reported in industries internationally where the risk was low before [21, 22], such as hydraulic fracturing and countertop production. Manufacturing of new products such as engineered stone has led to new cases of pneumoconiosis in the manufacturing sector [23, 24].

The occupations that showed significant association were mining industry occupations such as mining, metal plant operators, drivers and mining labourers. While electrotechnology, physical and engineering technicians along with machinery mechanics, blacksmiths and toolmakers were also significantly at risk in the mining industry, of interest is the majority of engineering professionals were not working in the mining industry indicating the occupation may be at risk, and further investigation is required. Recently an Australian report identified three workers: one engineer, an electrician and a fitter with coal workers’ pneumoconiosis supporting engineering as an occupation as at risk [25], while a 2006 Brazilian study in hospital cases of pneumoconiosis identified 14.5% of the cases occupations as blacksmiths [26].

There are two useful surveillance reports on pneumoconiosis in South Africa,the Pathaut report from the National Institute for Occupational Health and the Department of Minerals Occupational Health and Safety Reports. Both of these reports focus solely on the mining industry and provide the incidence of reported or diagnosed pneumoconiosis [27, 28]. The NIOH pathaut reports for 2013–2015 found 921 cases of pneumoconiosis this number is double those found in the mortality data for the same period, but this is not unexpected as these cases are found at autopsy after the cause of death has been certified. The DMR Mine Health and Safety Inspectorate annual report are annually for the financial year. For the year 2013 and 2014, 1714 and 1281 pneumoconiosis cases were reported by mines to the DMR, respectively.

The quality and amount of data limit the associations of the less commonly reported occupations and industries and diseases with minimal reported association with occupational disease. Completeness of the data is an important limitation. This should be emphasized at all levels within the departments responsible for data collection. The question of usual occupation is often misunderstood when completing the death registration form as can be seen by the number of pensioners and not economically active in the data set with pneumoconiosis. More training in this area for people responsible for the collection of information for the death certificate would improve occupation data and would provide valuable data for research and public health. The question on ‘usual occupation’ could be replaced by one or a combination of the following; longest held job or last held job for those who are retired. Although not as comprehensive as a complete job history, this would help improve the data available for surveillance. Another area for improvement is the SASCO 2012 edition of the standard classification of occupations; it needs to reflect the large number of elementary occupations in South Africa with more separate groups for these workers.

## Conclusions

The South African mortality data contains useful information on usual occupation and industry in the years 2013–2015. The identification of expected associations between pneumoconiosis and industry along with occupation demonstrates the benefits of using this data for occupational health investigations and surveillance despite the known limitations of the data. This analysis of pneumoconiosis deaths also demonstrates the value of requesting information on both usual occupation and industry. More often an exposure may be linked to the industry the person was employed in, rather than the job itself. A good example is salespeople working for the mining industry.

This analysis provided information on the expected industries and jobs but also indicated an at-risk group, engineers, electrotechnology technicians and mechanics not employed in the mining industry as at risk this warrants further investigation into these occupations for prevention efforts along with recommending risk assessments and possible controls for all mining industry jobs.

Thus, mortality data are a reasonable auxiliary source of information where there is no occupational disease surveillance system while taking into account the data limitations when interpreting findings.

## Data Availability

Freely available at Statistics South Africa online: http://www.statssa.gov.za/ (accessed 02/08/2017).

## Appendix

**Table:** Table 7 Coding categories in Stats Sa data for the years 2006-2015

	Major occupational categories (1 digit)	Location of armed forces category 0–13	Sub occupation Categories (2 digits)	Minor occupation (3 digits)	Number of Coding categories for Industry
2006	10 categories numbered 0–9	0	No	No	13
2007	10 categories numbered 0–9	0	no	No	13
2008	14 categories numbered 1–13	10	no	No	13
2009	11 categories numbered 0–9 and 99	0	31	No	13
2010	11 categories numbered 0–9 and 99	0	31	No	13
2011	11 categories numbered 0–9 and 99	0	43	No	150
2012	11 categories numbered 0–9 and 99	0	44	No	145
2013	11 categories numbered 0–9 and 99	5	44	Yes	138
2014	11 categories numbered 0–9 and 99	5	43	Yes	136
2015	10 categories numbered 0–9	5	43	Yes	137

## Abbreviations

CEO: Chief Executive Officer
CWP: Coal Workers Pneumoconiosis
DHA: Department of Home Affairs
DMR: Department of Mineral Resources
ISCO: International Standard Classification of Occupations
LFS: Labour Force Survey
MOR: Mortality Odds Ratio
NIOH: National Institute for Occupational Health
NIOSH: National Institute for Occupational Safety and Health
NOMS: National Occupational Mortality Surveillance
SASCO: South African Standard Classification of Occupations
TB: Tuberculosis
WHO: World Health Organisation
